# Low plasma citrulline levels are associated with acute respiratory distress syndrome in patients with severe sepsis

**DOI:** 10.1186/cc11934

**Published:** 2013-01-17

**Authors:** Lorraine B Ware, Jordan A Magarik, Nancy Wickersham, Gary Cunningham, Todd W Rice, Brian W Christman, Arthur P Wheeler, Gordon R Bernard, Marshall L Summar

**Affiliations:** 1 Department of Medicine, Division of Allergy, Pulmonary and Critical Care Medicine Vanderbilt University School of Medicine, T1218 MCN, 1161 21st Avenue S, Nashville, TN 37232-2650, USA; 2 Department of Pathology, Microbiology and Immunology, Vanderbilt University School of Medicine, 1161 21st Avenue S, Nashville, TN 37232, USA; 3 Department of Pediatrics, Division of Genetics and Metabolism, Children's National Medical Center, 111 Michigan Avenue, NW Washington, DC 20010, USA

## Abstract

**Introduction:**

The role of nitric oxide synthase (NOS) in the pathophysiology of acute respiratory distress syndrome (ARDS) is not well understood. Inducible NOS is upregulated during physiologic stress; however, if NOS substrate is insufficient then NOS can uncouple and switch from NO generation to production of damaging peroxynitrites. We hypothesized that NOS substrate levels are low in patients with severe sepsis and that low levels of the NOS substrate citrulline would be associated with end organ damage including ARDS in severe sepsis.

**Methods:**

Plasma citrulline, arginine and ornithine levels and nitrate/nitrite were measured at baseline in 135 patients with severe sepsis. ARDS was diagnosed by consensus definitions.

**Results:**

Plasma citrulline levels were below normal in all patients (median 9.2 uM, IQR 5.2 - 14.4) and were significantly lower in ARDS compared to the no ARDS group (6.0 (3.3 - 10.4) vs. 10.1 (6.2 - 16.6), *P *= 0.002). The rate of ARDS was 50% in the lowest citrulline quartile compared to 15% in the highest citrulline quartile (*P *= 0.002). In multivariable analyses, citrulline levels were associated with ARDS even after adjustment for covariates including severity of illness.

**Conclusions:**

In severe sepsis, levels of the NOS substrate citrulline are low and are associated with ARDS. Low NOS substrate levels have been shown in other disease states to lead to NOS uncoupling and oxidative injury suggesting a potential mechanism for the association between low citrulline and ARDS. Further studies are needed to determine whether citrulline supplementation could prevent the development of ARDS in patients with severe sepsis and to determine its role in NOS coupling and function.

## Introduction

The role of nitric oxide synthases (NOS) and their product nitric oxide (NO) in the pathophysiology of clinical acute respiratory distress syndrome (ARDS) is still not well understood. The signaling molecule NO can regulate a number of processes important in the pathophysiology of ARDS [1,2] including vascular tone, platelet aggregation, leukocyte adhesion, and mitochondrial oxygen consumption [3]. Production of NO is catalyzed by the three nitric oxide synthases (NOS), and all three isoforms of NOS (NOS-1, NOS-2 and NOS-3) are expressed in the lung.

**Figure 1:**
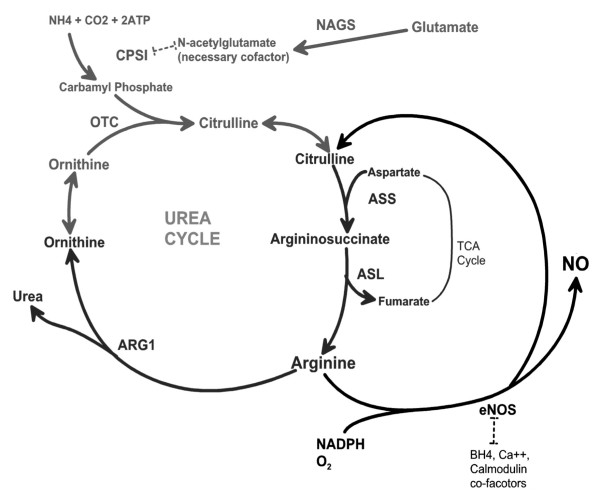
**Schematic of the integration of nitric oxide production with the urea cycle**. ARG1, arginase-1; CPSI, carbamyl phosphate synthase inhibitor; NADPH, nicotinamide adenine dinucleotide phosphate; NAGS, N-acetylglutamate synthase; NO, nitric oxide; OTC, ornithine transcarbamylase; TCA, tricarboxylic acid.

The proximal substrate for NO synthesis by NOS is L-arginine. L-arginine is synthesized primarily from the urea cycle intermediate L-citrulline by argininosuccinate synthase (ASS) and argininosuccinate lyase (ASL) (Figure 1). Experiments in our laboratories have shown that complexing of ASS and ASL with NOS in several tissues results in substrate channeling of citrulline through ASS and ASL to NOS to drive NO production [4]. In addition, we have recently observed that in human vascular endothelial cells, extracellular citrulline and not arginine, is the effective precursor of NO production. These findings suggest that circulating levels of citrulline may be more predictive of NOS function than arginine levels.

Circulating levels of L-citrulline are dependent on the proximal urea cycle function of the gut and secondarily the liver [5] and low levels of citrulline and arginine have been reported in adults [6,7] and children [8,9] with sepsis and other critical illness but have not been previously reported in ARDS. We hypothesized that the physiologic stress of sepsis would result in overall decreases in citrulline production to levels that could potentially lead to a fall in NO production in the lung, and potentiate the development of ARDS. We further hypothesized that arginine levels would not be associated with ARDS because of substrate channeling from citrulline to NO. To test this hypothesis, we measured plasma levels of the NO precursors citrulline and arginine, as well as the NO products nitrate and nitrite in 135 patients with severe sepsis enrolled in two randomized clinical trials of an anti-tumor necrosis factor antibody. For comparison, we also measured plasma levels of ornithine, an amino acid that is not a direct substrate for NOS but is an intermediate in the urea cycle.

## Materials and methods

### Patients

Of the 141 patients enrolled in two phase II randomized controlled studies of affinity-purified, anti-tumor necrosis factor-α ovine fab fragments (CytoFab) 135 had baseline plasma samples available for measurements and were included in this analysis. Plasma was drawn from patients as part of the clinical trial protocol at the time of enrollment. After collection, plasma was centrifuged to remove cells and the supernatant was stored in small aliquots at -80C until thawed for amino acid analysis. For inclusion in the clinical trials, documented or presumed infection, systemic inflammatory response syndrome (SIRS), and presence of shock or dysfunction of two other organs were required. Exclusion criteria have been previously published [10,11]. The institutional review board or independent ethics committee at each enrollment site approved the clinical trials [10,11], as did the Vanderbilt Institutional Review Board. Each participant or their surrogate provided informed consent to participate in the study. Patients were assessed for the presence of ARDS based on consensus definitions [12] on the day of clinical trial enrollment, which was also the day of baseline plasma collection. Clinical data including demographics, severity of illness scoring (Acute Physiology and Chronic Health Evaluation, APACHE II) [13], Brussels organ failure scores [14], laboratory values and outcomes were obtained from the study databases.

### Amino acid, nitrate and nitrite measurements

Plasma amino acid levels were measured by the Hitachi L-8800I high performance amino acid analyzer (Pleasanton, CA, USA). Plasma total nitrate and nitrite (NOx) were measured in duplicate by a Sievers 280I chemiluminescent nitric oxide analyzer (GE, Boulder, CO, USA).

### Statistical analysis

All statistical analysis was done using IBM SPSS Statistics Version 19.0 for Macintosh. Amino acid levels and NOx levels were not normally distributed and were compared between groups using the Mann-Whitney *U*-test. Correlations were assessed using Spearman rank correlation coefficients. Categorical variables were compared by chi square analysis or Fisher's exact test as appropriate. Multivariable logistic regression models were used to assess the potential confounding effects of clinical variables on the association of plasma citrulline levels with diagnosis of ARDS. Potential confounders were included in the model one at a time to assess the confounding effects of each variable on the relationship of citrulline to ARDS [15]. In logistic regression equations, citrulline was expressed as odds ratio (OR) per 5 uM increment. A *P*-value less than or equal to 0.05 was considered statistically significant.

## Results

### Patients

Patient characteristics are summarized in Table 1. Patients with ARDS at enrollment were younger and were more likely to be mechanically ventilated. They were more likely to have coagulation failure and less likely to have renal failure, but had a similar overall severity of illness as measured by the APACHE II score.

**Table 1:** Clinical characteristics of 135 patients with severe sepsis

Characteristic	All patients^1^	ARDS *N *= 44	No ARDS *N *= 91	*P*-value^2^
Age, years	55 ± 16	49 ± 17	57 ± 16	0.011
Male, %	54	50	65	0.10
Caucasian, %	73	82	69	0.12
APACHE II score	24 ± 7	24 ± 7	24 ± 7	0.97
WBC, × 10^3^	17.6 ± 12.0	17.0 ± 11.7	18.0 ± 12.1	0.66
Creatinine, mg/dL	2.4 ± 2.1	2.0 ± 2.3	2.6 ± 2.0	0.14
PaO_2_/FiO_2 _ratio	155 ± 88	102 ± 51	184 ± 91	< 0.001
Mechanically ventilated at enrollment, %	84	95	79	0.014
Liver failure, %	23	22	24	0.84
Renal failure, %	41	25	41	0.01
Coagulation failure, %	28	46	20	0.002
Mortality at 28 days, %	36	39	34	0.60

**^1^**Data are mean ± SD or percent of patients as indicated. ^2^*P*-value for comparison of patients with ARDS or without ARDS. APACHE II, Acute Physiology and Chronic Health Evaluation; WBC, white blood cells; PaO_2_/FiO_2_, arterial to inspired oxygen ratio.

### Amino acid levels and ARDS

The median plasma level of citrulline was very low at 9.2 uM (interquartile range (IQR) 5.2 to 14.4) compared to a normal range of 40 ± 10 uM in healthy adults [16]. The median plasma level of arginine was also low at 22.7 uM (IQR 12.8 to 37.9) compared to a normal range of 27 to 80 uM. The median level of ornithine was 22.7 uM (IQR 14.3 to 39.0), which was within the normal range of 13 to 64 uM.

**Figure 2:**
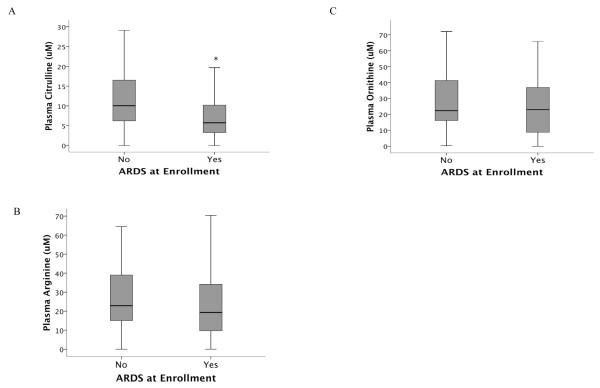
**Box plot summary of plasma levels of citrulline (panel A), arginine (panel B) and ornithine (panel C) in 135 patients with severe sepsis**. Citrulline levels were significantly lower in patients with acute respiratory distress syndrome (ARDS) compared to patients without ARDS. Horizontal line represents median, box encompasses 25^th ^to75^th ^percentile, error bars encompass 10^th ^to 90^th ^percentile. *P = 0.002.

**Figure 3:**
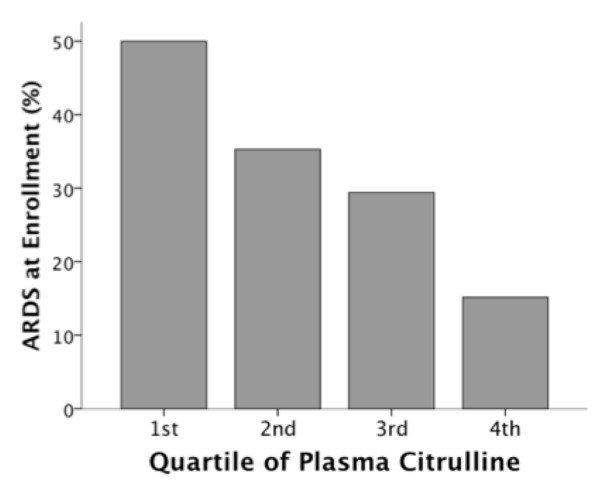
**Incidence of acute respiratory distress syndrome (ARDS) by quartile of plasma citrulline level**. Plasma citrulline levels in each quartile were: 1st quartile 0 to 5.12 uM, 2^nd ^quartile 5.16 to 8.56 uM, 3^rd ^quartile 9.15 to 14.18 uM, 4^th ^quartile 14.35 to 50.07 uM. Patients with the lowest plasma citrulline level (1^st ^quartile) had the highest percentage of patients with ARDS. *P *= 0.002 for trend across quartiles.

Amino acid levels are compared between patients with and without ARDS in Table 2. Plasma levels of citrulline were significantly lower in patients with ARDS at enrollment compared to patients without ARDS (Figure 2a). By contrast, plasma levels of ornithine and arginine did not differ significantly between patients with and without ARDS (Figures 2b and 2c). Among patients in the lowest quartile of plasma citrulline level, the incidence of ARDS was 50% compared to an incidence of 15% in the highest quartile of plasma citrulline (*P *= 0.002 for trend across quartiles) (Figure 3).

**Table 2:** Comparison of baseline plasma amino acid and NOx levels in patients with and without ARDS

Analyte	ARDS^1^ *n *= 44	No ARDS *n *= 91	*P*-value
Citrulline, uM	6.0 (3.3 - 10.4)	10.1 (6.2 - 16.6)	0.002
Arginine, uM	19.3 (9.6 - 34.5)	22.9 (14.7 - 40.0)	0.18
Ornithine, uM	23.0 (8.5 - 37.1)	22.3 (16.0 - 43.2)	0.22
NOx	53.8 (24.2 - 92.3)	49.8 (34.5 - 80.9)	0.89

**^1^**Data presented as median (IQR). ARDS, acute respiratory distress syndrome; NOx, total nitrate and nitrite.

### Amino acid levels and non-pulmonary organ failure

Amino acid levels were not different between patients with and without renal failure (median 9.4, IQR 5.0 to 12.7 vs. 8.3 uM, IQR 5.2 to 15.1; *P *= 0.68). There were also no differences in amino acid levels between patients with and without cardiovascular failure (shock) and liver failure as defined by the Brussels organ failure scoring system. However, there was a trend towards lower citrulline levels in patients with coagulation failure (thrombocytopenia) (*P *= 0.060). Plasma NOx levels were significantly higher in patients with renal failure (median 70.6 Um, IQR 41.4 to 101.1 vs. 40.6 uM, IQR 22.6 to 72.1; *P *< 0.001), and plasma NOx levels were significantly associated with serum creatinine measurements (*r *= 0.48; *P *< 0.001).

### Multivariable analysis

The association of plasma citrulline levels with ARDS was assessed after adjustment for potential confounding variables in multivariable analyses. The unadjusted OR for ARDS associated with a 5 uM decrease in plasma citrulline level was 1.50 (95% CI, 1.14, 1.98; *P *= 0.004). Adjustment for other covariates including age, gender, ethnicity, APACHE II level, and presence of shock did not alter this relationship (Table 3).

**Table 3:** Multivariable analyses of association between plasma citrulline levels and ARDS in patients with severe sepsis

Variable	Odds ratio per 5 uM decrease in plasma citrulline (95% CI)	*P*-value
Unadjusted	1.50 (1.14, 1.98)	0.004
Adjusted for:		
Age	1.43 (1.08, 1.89)	0.012
Gender	1.52 (1.14, 2.02)	0.004
Ethnicity	1.50 (1.14, 1.97)	0.004
APACHE II score	1.50 (1.14, 1.98)	0.004
Presence of shock	1.51 (1.14, 1.99)	0.004

ARDS, acute respiratory distress syndrome; APACHE II, Acute Physiology and Chronic Health Evaluation.

### Clinical outcomes

In patients with ARDS, amino acid levels were not significantly associated with mortality (Table 4) or ventilator-free days (not shown).

**Table 4:** Comparison of plasma amino acids and biomarkers of lung injury by 28-day mortality in patients with ARDS (*n *= 44)

Analyte	Lived^1^ *n *= 27	Died *n *= 17	*P*-value
Citrulline	5.7 (3.6 - 9.8)	7.5 (1.9 - 12.6)	0.56
Arginine (uM)	19.9 (10.3 - 32.8)	18.8 (7.8 - 37.8)	0.88
Ornithine (uM)	23.2 (11.5 - 34.9)	20.6 (5.3 - 41.0)	0.74
NOx	34.8 (23.4 - 72.7)	60 (42.2 - 124.2)	0.06

**^1^**Data as presented as median (IQR). ARDS, acute respiratory distress syndrome; NOx, total nitrate and nitrite.

## Discussion

We hypothesized that the physiologic stress of sepsis would result in decreased citrulline production and that lower circulating levels of citrulline would be associated with the occurrence of ARDS. We observed markedly low plasma levels of citrulline in all patients with severe sepsis, and a significant association between lower citrulline levels and the occurrence of ARDS in sepsis. Furthermore, lower levels of plasma citrulline were independently associated with a diagnosis of ARDS, even when controlling for measures of severity of illness. The finding of a strong association between low citrulline levels and presence of ARDS in sepsis patients suggests that NOS substrate deficiency might play a role in the pathogenesis of ARDS.

**Figure 4:**
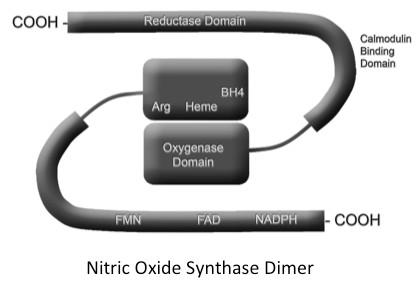
**Schematic of a nitric oxide synthase (NOS) dimer with both the oxygenase and reductase domains shown**. Coupling of electron transfer between these domains leads to synthesis of nitric oxide (NO). When uncoupled, NOS preferentially catalyzes the production of superoxide ion from reduction of molecular oxygen rather than NO. Uncoupling of NOS can be caused by oxidative stress, ischemia reperfusion and substrate deficiency. Arg, arginine; FMN, flavin mononucleotide binding domain; FAD, flavin adenine dinucleotide binding domain; NADPH, nicotinamide adenine dinucleotide phosphate.

NOS are modular enzymes with both a reductase and oxygenase domain (Figure 4). Coupling of electron transfer between these domains leads to synthesis of NO. When uncoupled, NOS preferentially catalyzes the production of superoxide ion from reduction of molecular oxygen rather than NO [17]. Uncoupling of NOS can be caused by oxidative stress [18], ischemia reperfusion [19] and substrate deficiency [17]. In the current study, substrate deficiency of citrulline would be expected to lead to insufficient precursor in the ASS-ASL-NOS system and uncoupling of NOS. This could be an important mechanism contributing to the pathogenesis of ARDS, since NOS uncoupling leads to an increase in superoxide production and oxidant stress. Chronic uncoupling of NOS is important in a variety of disease processes including diabetes [3], hypertension [3] and diastolic dysfunction [20].

Although we hypothesized that substrate deficiency of citrulline would lead to decreased NO production, it was not possible to measure NO production prospectively in the current study. We did measure plasma levels of NOx, the stable byproducts of NO production. Plasma levels of citrulline and arginine were not associated with plasma levels of NOx, nor were NOx levels associated with the presence of ARDS. Although one might predict that substrate deficiency would lead to a fall in NO production, and a resultant fall in plasma NOx levels, others have reported that circulating NOx levels are strongly modulated by renal excretion of NOx, which is impaired in the setting of renal insufficiency [21]. Concordant with these findings, plasma NOx levels in our study were strongly and inversely associated with serum creatinine measurements, and were significantly higher in patients with renal failure. In a separate cohort, we previously reported that low levels of urine nitric oxide species (NOx) are an independent predictor of mortality in patients with ARDS [22]. Unfortunately, no urine was collected in the present study for analysis of urine NOx species.

To our knowledge, there have not been any prior studies of urea cycle products in clinical ARDS, although there have been several small studies in patients with sepsis. Freund *et al. *[23] measured plasma amino acid levels in 25 patients with sepsis and found that low arginine levels were associated with mortality; citrulline was not measured in that study. Druml and colleagues [24] also reported low levels of plasma arginine in nine patients with sepsis. In 59 critically ill children, plasma arginine and citrulline levels were lower in patients with sepsis or trauma compared to patients with viral disease and were inversely associated with severity of inflammation as indicated by the plasma C-reactive protein concentration [8]. Several studies have examined arginine and citrulline flux in critical illness and sepsis. Plasma arginine levels and arginine production have been found to be low in six adults with septic shock compared to healthy controls [21]. In 13 adults with sepsis, plasma arginine and citrulline levels were found to be low compared to controls, as was the rate of citrulline flux [9]. Luiking *et al. *reported similar findings of very low citrulline levels and low rates of citrulline production in 10 patients with septic shock and concluded that the fall in citrulline production was driven by diminished *de novo *arginine and NO production [7]. The current findings of low plasma levels of citrulline and arginine in patients with severe sepsis are concordant with these prior studies and provide additional new information regarding the independent association of low citrulline levels with development of ARDS in severe sepsis. In light of our recent report of ASS-ASL-NOS complex formation and substrate channeling [4], citrulline substrate availability is likely to be the primary external factor affecting the ability of cells to maintain coupling of NOS. Furthermore, citrulline enters the cell with relatively little competition through neutral amino transport, while arginine uses dibasic amino acid transport, and we detect very little free arginine inside cells [25], findings that also favor a primary role for citrulline availability in regulation of NOS coupling.

Although it was not possible in this observational study to determine the mechanisms that lead to low citrulline levels in this critically ill cohort of patients with severe sepsis, there are several possible mechanisms. Decreased nutritional intake is common in the early stages of critical illness and could be a potential mechanism leading to low substrate levels for citrulline production. However, levels of ornithine and other amino acids such as glycine and glutamine (data not shown) were in the normal range, suggesting that decreased nutritional intake is not the primary driver of low citrulline and arginine levels in this study. Gut enterocytes are an important source of citrulline production that can be injured in critical illness. Indeed, plasma citrulline levels have been proposed as a functional indicator of small bowel enterocyte mass [26]. Thus, decreased enterocyte production is one potential mechanism of low citrulline levels that may be relevant in both sepsis and ARDS [27]. Although we did not measure arginase activity in this study, increases in arginase activity could also contribute to low circulating citrulline levels since arginase and NOS compete for arginine substrate. Arginase activity can increase in the setting of sepsis and oxidative stress [28]. However, in a shock model in rats, arginase in the lung was downregulated, arguing against arginase upregulation in the lung as a major determinant of local levels in the lung [29].

The association of low levels of plasma citrulline with development of ARDS in patients with severe sepsis might have implications for the prevention and/or treatment of ARDS. Citrulline repletion is already being tested in other clinical settings that are associated with NOS substrate deficiency. In pulmonary hypertension after congenital heart surgery, low levels of citrulline have been shown to predict reduced NO production and poor clinical outcomes [30]. In an early phase clinical trial, intravenous citrulline supplementation was safe and well tolerated in children undergoing congenital heart surgery with cardiopulmonary bypass, and led to increases in plasma NOx and arginine levels as well as a reduction in post-operative pulmonary hypertension, without any evidence of systemic changes in blood pressure [31]. A larger clinical trial in children undergoing cardiac surgery with cardiopulmonary bypass is ongoing.

This study has several strengths. A major strength is the size of the study with 135 critically ill patients, which to our knowledge is the largest study of NOS substrate levels in critical illness to date. Because of the large size of the study we were able to test the association of NOS substrate levels with other organ failures and clinical outcomes in addition to ARDS. This study also has limitations. First, although the association between low levels of citrulline and ARDS was robust, it provides only indirect evidence that substrate deficiency leading to NOS uncoupling is important in the pathophysiology of ARDS; we were not able to directly measure either NO or superoxide production in this study, and the plasma NOx measures appeared to be modulated by renal failure. In addition, ARDS could be a cause rather than a consequence of low substrate levels. A second limitation is that we did not measure tetrahydrobiopterin (BH_4_) levels. BH_4 _is a NOS cofactor that is normally bound to the NOS oxygenase domain [19]. BH_4 _levels can be reduced in the setting of oxidative stress [18] and ischemia reperfusion [19]. However, like NOx, circulating BH_4 _levels are affected by renal failure [32], and it is not clear that circulating levels are a good indicator of potential BH_4 _effects on NOS. A final limitation is that the measurements were made at only a single point in time. In future studies it would be helpful to determine changes in citrulline and arginine levels over time in association with development of ARDS.

## Conclusions

In summary, plasma citrulline levels are low in patients with severe sepsis and low levels are associated with the presence of ARDS. Further studies are needed to determine whether citrulline supplementation could prevent the development of ARDS in patients with severe sepsis.

## Key messages

• Circulating levels of citrulline, the primary substrate for NOS, are low in patients with severe sepsis.

• Low levels of circulating citrulline are associated with development of ARDS in patients with severe sepsis.

• Prospective studies are needed to determine whether citrulline supplementation could prevent the development of ARDS in patients with severe sepsis.

## Abbreviations

APACHE II: Acute Physiology and Chronic Health Evaluation; ARDS: acute respiratory distress syndrome; ASL: argininosuccinate lyase; ASS: arginonosuccinate synthase; BH_4_: tetrahydrobiopterin; IQR: interquartile range; NO: nitric oxide; NOS: nitric oxide synthase; NOx: total nitrate and nitrite; OR: odds ratio; SIRS: systemic inflammatory response syndrome.

## Competing interests

The authors declare they have no competing interests. Vanderbilt University has applied for and received patents for the therapeutic use of intravenous citrulline.

## Authors' contributions

LW conceived and designed the study, analyzed the data, and wrote and edited the manuscript. JM, NW and GC performed the amino acid measurements and edited the manuscript. TR, BC, AW, and GB designed the study, enrolled patients, analyzed data and edited the manuscript. MS conceived and designed the study, analyzed the data and edited the manuscript. All authors read and approved the final manuscript.

## References

[B1] Ware LB Matthay MA Medical progress: The acute respiratory distress syndrome N Engl J Med 2000 17 1334 1349 10.1056/NEJM200005043421806 10793167

[B2] Ware LB Pathophysiology of acute lung injury and the acute respiratory distress syndrome Semin Respir Crit Care Med 2006 17 337 349 10.1055/s-2006-948288 16909368

[B3] Luiking YC Engelen MP Deutz NE Regulation of nitric oxide production in health and disease Curr Opin Clin Nutr Metab Care 2010 17 97 104 10.1097/MCO.0b013e328332f99d 19841582 PMC2953417

[B4] Erez A Nagamani SC Shchelochkov OA Premkumar MH Campeau PM Chen Y Garg HK Li L Mian A Bertin TK Black JO Zeng H Tang Y Reddy AK Summar M O'Brien WE Harrison DG Mitch WE Marini JC Aschner JL Bryan NS Lee B Requirement of argininosuccinate lyase for systemic nitric oxide production Nat Med 2011 17 1619 1626 10.1038/nm.2544 22081021 PMC3348956

[B5] Neill MA Aschner J Barr F Summar ML Quantitative RT-PCR comparison of the urea and nitric oxide cycle gene transcripts in adult human tissues Mol Genet Metab 2009 17 121 127 10.1016/j.ymgme.2009.02.009 19345634 PMC2680466

[B6] Freund H Atamian S Holroyde J Fischer JE Plasma amino acids as predictors of the severity and outcome of sepsis Ann Surg 1979 17 571 576 10.1097/00000658-197911000-00003 389183 PMC1344534

[B7] Luiking YC Poeze M Ramsay G Deutz NE Reduced citrulline production in sepsis is related to diminished de novo arginine and nitric oxide production Am J Clin Nutr 2009 17 142 152 19056593 10.3945/ajcn.2007.25765

[B8] van Waardenburg DA de Betue CT Luiking YC Engel M Deutz NE Plasma arginine and citrulline concentrations in critically ill children: strong relation with inflammation Am J Clin Nutr 2007 17 1438 1444 17991657 10.1093/ajcn/86.5.1438

[B9] Kao CC Bandi V Guntupalli KK Wu M Castillo L Jahoor F Arginine, citrulline and nitric oxide metabolism in sepsis Clin Sci (Lond) 2009 17 23 30 10.1042/CS20080444 19105791

[B10] Wheeler A Dupont W Edens T Higgins S Wickersham N Bernard G Impact of polyclonal anti-TNF Fab frabments on plasma cytokines in sepsis [Abstract] Am J Respir Crit Care Med 1999 17 A263

[B11] Rice TW Wheeler AP Morris PE Paz HL Russell JA Edens TR Bernard GR Safety and efficacy of affinity-purified, anti-tumor necrosis factor-alpha, ovine fab for injection (CytoFab) in severe sepsis Crit Care Med 2006 17 2271 2281 10.1097/01.CCM.0000230385.82679.34 16810105

[B12] Bernard GR Artigas A Brigham KL Carlet J Falke K Hudson L Lamy M Legall JR Morris A Spragg R the Consensus Committee The American-European Consensus Conference on ARDS. Definitions, mechanisms, relevant outcomes, and clinical trial coordination Am J Respir Crit Care Med 1994 17 818 824 7509706 10.1164/ajrccm.149.3.7509706

[B13] Knaus WA Draper EA Wagner DP Zimmerman JE APACHE II: a severity of disease classification system Crit Care Med 1985 17 818 829 10.1097/00003246-198510000-00009 3928249

[B14] Bernard G The Brussels Score Sepsis 1997 17 43 44 10.1023/A:1009711301483

[B15] Maldonado G Greenland S Simulation study of confounder-selection strategies Am J Epidemiol 1993 17 923 936 8256780 10.1093/oxfordjournals.aje.a116813

[B16] Crenn P Vahedi K Lavergne-Slove A Cynober L Matuchansky C Messing B Plasma citrulline: A marker of enterocyte mass in villous atrophy-associated small bowel disease Gastroenterology 2003 17 1210 1219 10.1016/S0016-5085(03)00170-7 12730862

[B17] Forstermann U Munzel T Endothelial nitric oxide synthase in vascular disease: from marvel to menace Circulation 2006 17 1708 1714 10.1161/CIRCULATIONAHA.105.602532 16585403

[B18] Landmesser U Dikalov S Price SR McCann L Fukai T Holland SM Mitch WE Harrison DG Oxidation of tetrahydrobiopterin leads to uncoupling of endothelial cell nitric oxide synthase in hypertension J Clin Invest 2003 17 1201 1209 12697739 10.1172/JCI14172 PMC152929

[B19] Verma S Maitland A Weisel RD Fedak PW Pomroy NC Li SH Mickle DA Li RK Rao V Novel cardioprotective effects of tetrahydrobiopterin after anoxia and reoxygenation: Identifying cellular targets for pharmacologic manipulation J Thorac Cardiovasc Surg 2002 17 1074 1083 10.1067/mtc.2002.121687 12063453

[B20] Silberman GA Fan TH Liu H Jiao Z Xiao HD Lovelock JD Boulden BM Widder J Fredd S Bernstein KE Wolska BM Dikalov S Harrison DG Dudley SC Jr Uncoupled cardiac nitric oxide synthase mediates diastolic dysfunction Circulation 2010 17 519 528 10.1161/CIRCULATIONAHA.109.883777 20083682 PMC2819317

[B21] Villalpando S Gopal J Balasubramanyam A Bandi VP Guntupalli K Jahoor F In vivo arginine production and intravascular nitric oxide synthesis in hypotensive sepsis Am J Clin Nutr 2006 17 197 203 16825696 10.1093/ajcn/84.1.197

[B22] McClintock DE Ware LB Eisner MD Wickersham N Thompson BT Matthay MA Higher urine nitric oxide is associated with improved outcomes in patients with acute lung injury Am J Respir Crit Care Med 2007 17 256 262 17082495 10.1164/rccm.200607-947OC PMC1899263

[B23] Freund H Atamian S Fischer JE Chromium deficiency during total parenteral nutrition Jama 1979 17 496 498 10.1001/jama.1979.03290310036012 104057

[B24] Druml W Heinzel G Kleinberger G Amino acid kinetics in patients with sepsis Am J Clin Nutr 2001 17 908 913 11333844 10.1093/ajcn/73.5.908

[B25] Fike CD Sidoryk-Wegrzynowicz M Aschner M Summar M Prince LS Cunningham G Kaplowitz M Zhang Y Aschner JL Prolonged hypoxia augments L-citrulline transport by System A in the newborn piglet pulmonary circulation Cardiovasc Res 2012 17 375 384 10.1093/cvr/cvs186 22673370 PMC3400357

[B26] Peters JH Beishuizen A Keur MB Dobrowolski L Wierdsma NJ van Bodegraven AA Assessment of small bowel function in critical illness: potential role of citrulline metabolism J Intensive Care Med 2011 17 105 110 10.1177/0885066610387998 21464064

[B27] Crenn P Messing B Cynober L Citrulline as a biomarker of intestinal failure due to enterocyte mass reduction Clin Nutr 2008 17 328 339 10.1016/j.clnu.2008.02.005 18440672

[B28] Bune AJ Shergill JK Cammack R Cook HT L-arginine depletion by arginase reduces nitric oxide production in endotoxic shock: an electron paramagnetic resonance study FEBS Lett 1995 17 127 130 10.1016/0014-5793(95)00495-U 7789529

[B29] Carraway MS Piantadosi CA Jenkinson CP Huang YC Differential expression of arginase and iNOS in the lung in sepsis Exp Lung Res 1998 17 253 268 10.3109/01902149809041533 9635249

[B30] Barr FE Beverley H VanHook K Cermak E Christian K Drinkwater D Dyer K Raggio NT Moore JH Christman B Summar M Effect of cardiopulmonary bypass on urea cycle intermediates and nitric oxide levels after congenital heart surgery J Pediatr 2003 17 26 30 10.1067/mpd.2003.mpd0311 12520250

[B31] Barr FE Tirona RG Taylor MB Rice G Arnold J Cunningham G Smith HA Campbell A Canter JA Christian KG Drinkwater DC Scholl F Kavanaugh-McHugh A Summar ML Pharmacokinetics and safety of intravenously administered citrulline in children undergoing congenital heart surgery: potential therapy for postoperative pulmonary hypertension J Thorac Cardiovasc Surg 2007 17 319 326 10.1016/j.jtcvs.2007.02.043 17662768

[B32] Galley HF Le Cras AE Yassen K Grant IS Webster NR Circulating tetrahydrobiopterin concentrations in patients with septic shock Br J Anaesth 2001 17 578 580 10.1093/bja/86.4.578 11573638

